# Microphysiological modeling of the Blood-CSF barrier for biopharmaceutical transport and sequestration: evaluation of PEG-induced accumulation and vacuolation in the choroid plexus epithelium

**DOI:** 10.3389/fphar.2026.1861778

**Published:** 2026-08-07

**Authors:** Jacquelyn Ann Brown, Monika G. Judge, David K. Schaffer, Dillon C. Gavlock, Armando Irizarry Rovira, Steven K. Engle, Thomas K. Baker, John P. Wikswo

**Affiliations:** 1 Organ Pathobiology and Therapeutics Institute, University of Pittsburgh, Pittsburgh, PA, United States; 2 Department of Computational and Systems Biology, School of Medicine, University of Pittsburgh, Pittsburgh, PA, United States; 3 Department of Physics and Astronomy, Vanderbilt University, Nashville, TN, United States; 4 Vanderbilt Institute for Integrative Biosystems Research and Education, Vanderbilt University, Nashville, TN, United States; 5 Lilly Research Laboratories, A Division of Eli Lilly and Company, Indianapolis, IN, United States; 6 Department of Biomedical Engineering, Vanderbilt University, Nashville, TN, United States; 7 Department of Molecular Physiology and Biophysics, Vanderbilt University, Nashville, TN, United States

**Keywords:** blood-brain barrier (BBB), blood-CSF barrier, central nervous system (CNS), microphysiological system (MPS), polyethylene glycols (PEG)

## Abstract

**Background:**

It has long been established that the central nervous system (CNS) is a highly privileged space, with the blood-brain barrier (BBB) acting as the gatekeeper that allows or denies access to the brain by nutrients, drugs, and toxins. The BBB, however, is not the only barrier at play. The barrier between blood and cerebrospinal fluid (CSF) also has a critical role in guarding and feeding the CNS, but despite its importance, the blood-CSF barrier (BCSFB) remains relatively unexplored compared to the BBB, particularly with regard to models that do not rely on animals.

**Methods:**

Given the lack of physiologically relevant *in vitro* models and the divergence between humans and animal models, we created an organ-on-chip/microphysiological system to model the human BCSFB (hBCSFB) that can act as a new approach methodology (NAM) platform for interrogating physiological functions, evaluating drug delivery technologies and xenobiotic transport, and assessing the safety, efficacy, and mechanism of action of biopharmaceuticals.

**Results:**

By leveraging advanced microfluidic organ chip design and gravity perfusion, we have generated a hBCSFB organ chip that recapitulates many of the physiologically relevant characteristics of this barrier, including junctional protein expression, permeability less than 1.55e-5 cm/s, transcytosis that showed dose-dependent accumulation of 10%–35%, and selective transport that was both statistically significant (p ≤ 0.05) and reproducible.

**Conclusion:**

Having achieved these benchmarks and validated their reproducibility, this work provides a useful *in vitro* system optimized for the qualitative and quantitative study of pharmacological and toxicological interactions involving the hBCSFB.

## Background

The functional unit of the blood-cerebrospinal fluid barrier (BCSFB) consists of a capillary surrounded by a layer of differentiated ependymal epithelial cells. Unlike the capillaries of the blood-brain barrier (BBB), those of the choroid plexus are fenestrated and lack tight junctions, so the endothelium does not act as a selective barrier to small molecules; instead, barrier function at the BCSFB is provided by epithelial cells joined by tight junctions (Johanson et al., 2011; Laterra et al., 1999; Liddelow, 2015; Lun et al., 2015). Functionally, the choroid plexus epithelium represents a key site for selective transport into the brain, facilitating exchange of essential compounds such as calcium (Ca^2+^), insulin-like growth factor 2 (IGF-2), and transthyretin, which carries thyroxine and retinol into the central nervous system (CNS) (Stepien and Huttner, 2019). The BCSFB and BBB also differ mechanically: shear force at the BCSFB is considerably lower (∼0.1–0.7 dyn/cm^2^) (Chatterjee et al., 2020) than the shear force observed in the microcapillaries of the BBB (∼5–23 dyn/cm^2^) (Choublier et al., 2022), a difference largely attributed to the fenestrations present in the BCSFB but absent in the BBB (Kadry et al., 2020). More broadly, although the mechanisms underlying differential transport across the BBB versus the BCSFB remain poorly understood, the two barriers are distinguished by passive permeability, tight junction protein expression, and transporter profiles. The BCSFB is generally more permeable than the BBB (Dabbagh et al., 2022) and supports distinct physiological roles; its disruption has been implicated in altered CSF homeostasis, neuroendocrine signaling, excretory function, and immune surveillance, contributing to diverse brain pathologies (Kant et al., 2018).

Given the BCSFB’s essential role in CNS function and its growing implication in neurodegenerative disease, there is an urgent need for physiologically relevant new approach methodologies (NAMs) and models. Such systems are critical for probing barrier physiology, evaluating drug delivery strategies, and assessing the safety, efficacy, and mechanism of action of biopharmaceuticals. Current nonclinical approaches to studying the choroid plexus include animal models, conventional two-dimensional cultures, and organoid/MPS systems, each with distinct strengths and limitations.

Animal models: Studies of BCSFB development, structure, and function in animals have greatly informed our general understanding of the barrier across species and enabled valuable inferences about the human BCSFB (hBCSFB) (Dabbagh et al., 2022). However, known species-specific differences in BCSFB structure and function (Saunders et al., 2023) mean that animal studies can yield conflicting findings, complicating interpretation of therapeutic responses. For example, given the growing use of PEGylation to tune the solubility and pharmacokinetics of biopharmaceuticals (Santhanakrishnan et al., 2024), it is notable that high-molecular-weight polyethylene glycols (PEGs) are widely reported not to efficiently cross the BCSFB, yet substantial evidence indicates their accumulation in the choroid plexus epithelium, where they are associated with vacuolation that increases with PEG size (Fang et al., 2022; Fletcher et al., 2019; Rudmann et al., 2013). This raises critical questions: if high-molecular-weight PEGs are not transported or transcytosed across the BCSFB, how do they accumulate within epithelial cells, and does their accumulation affect barrier function? Together with *in vivo* data (McClung et al., 1990), these findings support the use of compounds spanning a range of molecular weights as strong candidates for probing BCSFB function in drug development. This is particularly important given the potential for species-specific effects of *in vivo* animal models that may not accurately replicate human BCSFB properties, may yield conflicting results, and may have limited translational value, especially with respect to molecular transport across the barrier (Stepien and Huttner, 2019; Saunders et al., 2023; Wakai, 1981). Collectively, these factors raise real questions about how confidently animal models can predict the hBCSFB’s response to, or transport of, xenobiotics such as biotherapeutics.

Two-dimensional and organoid systems: Traditional two-dimensional *in vitro* systems have provided useful insights but suffer from several well-recognized limitations: lack of three-dimensional architecture, excessive paracellular leakage, absence of endothelial-epithelial interaction, no shear stress to induce polarization, no media flow to clear locally secreted cytokines and growth factors that can alter cellular phenotype, and restricted capacity for real-time evaluation. These limitations are compounded by the fact that vascular and epithelial compartments require different culture media *in vivo*, whereas conventional systems typically rely on a single medium, often leading to dysregulated barrier behavior when serum, needed for endothelial cell survival, is introduced (Neal et al., 2021). Organoid models improve upon cellular complexity and spatial organization and have been used to demonstrate CSF production (Pellegrini et al., 2020) and, more recently, to study *Streptococcus suis* infection and its translocation across the BCSFB (Zhao et al., 2025). The utility of organoid-based models is clear, but as with any model system, there are areas where other approaches may excel, including fluidic shear stress and cytokine/growth factor washout, (8) lack of vascular integration, the challenges of quantifying barrier transport, and batch-to-batch variability (Bryniarski et al., 2020; von Maydell and Jorfi, 2021).

Microphysiological systems (MPS): Organ-on-a-chip microphysiological systems address many of these gaps by incorporating perfusion, compartmentalization, and dynamic monitoring, yet most existing platforms have been designed for the BBB (Brown et al., 2016; Brown et al., 2015; Brown et al., 2014; Prabhakarpandian et al., 2013) and remain technically demanding to operate. Newer MPS models targeting the BCSFB specifically hold considerable promise (Zhou et al., 2023). As with any system, though, there are trade-offs in which endpoints a given platform can support: thinner platforms are typically better suited to image-based analysis but lack the biomass needed for many secreted-factor assays, while thicker bioreactors offer greater biomass at the cost of imaging quality. Provided the underlying physiology is robust and biologically relevant, there is no single “correct” platform: the key consideration is choosing the system best suited to the specific scientific question and endpoints of interest (Schwerk and Schroten, 2024).

While animal models remain valuable, the limitations of the current *in vivo* alternatives underscore the need for human-relevant NAMs. In line with NIH and FDA mandates for NAM adoption (Zushin et al., 2023), advanced MPS models of the BCSFB are essential to resolve key mechanistic uncertainties, uncover barrier-specific physiology, elucidate disease mechanisms, and accelerate the development of targeted CNS therapeutics (Logan et al., 2019; Faley et al., 2025).

## Materials and methods

### Gravity chip design and assembly

**FIGURE 1:**
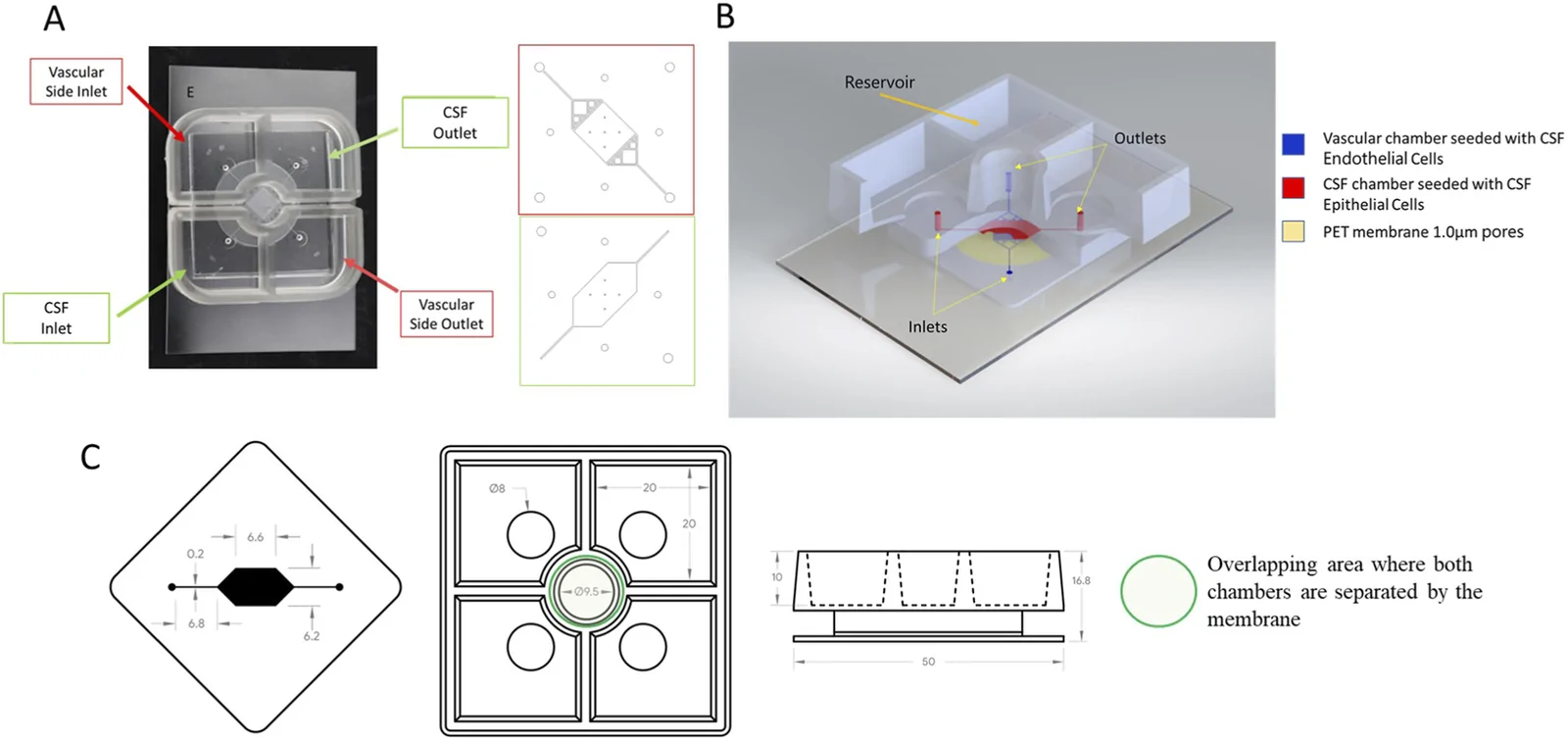
Design and layout of gravity-perfused BCSFB MPS. **(A)** Photograph of a BCSFB gravity chip and schematic of the 2 cell culture chambers that comprise the BCSFB MPS. **(B)** 3D rendering of the BCSFB gravity chip. Blue is the vascular chamber where the choroid plexus endothelial cells are seeded. Red is the brain/CSF chamber seeded with CSF epithelial cells. Yellow is the polyethylene terephthalate (PET) membrane with 1 µm track-etched pores. **(C)** Detailed dimensions of the gravity BCSFB chip in mm.

Given the practical challenges of perfusing organ chips with either syringe pumps, sealed pressurized reservoirs, or in-line peristaltic pumps, we have created a two-compartment, gravity-perfused barrier chip where each chamber is perfused by a height difference between a supply and a collection reservoir. The volume of these reservoirs is large, yet low profile, to ensure that the high flow rates required for endothelial polarization by flow-induced shear forces can be maintained for 24 h, with a daily removal of media from the collection reservoir and replenishment of media in the supply reservoir being straightforward and without the risk of introducing cell-disrupting bubbles into either chamber. We used a variety of soft lithographic, molding, and bonding techniques to create the three-layer device shown in Figures 1A,B, comprising reservoir, CSF, and vascular layers, with the latter two separated by a semipermeable membrane that supports the actual BCSFB. Chip dimensions are shown in Figure 1C. Wall shear stress in a microfluidic device with a wide rectangular channel is calculated using τ _wall=6_ $\frac{6nQ}{Wh^{2}}$ , where dynamic viscosity (η), flow rate (Q), channel height (h), and channel width (W) are expressed in CGS units of poise, cm3/s, and cm, respectively. The resulting value represents the wall shear stress in dyn/cm^2^. At a flow rate of 5 μL/min that decreases over the 24 h before being refreshed, calculated shear stress is between 0.1–0.7 dyn/cm^2^. We previously described a gravity-perfused blood-brain barrier chip (Boghdeh et al., 2022) that is the basis for the BCSFB chips used in this study (Faley et al., 2025). Flow is driven by the hydrostatic pressure created by the inlet reservoirs being at a higher elevation than the outlet; thus, the media flows downhill to create perfusion driven by gravity as opposed to a pump-perfused device, where the syringe pump provides constant positive pressure to produce flow.

### BCSFB cells and culture

Primary cell culture was carried out as described previously (Liddelow, 2015; Lun et al., 2015). Primary human CSF endothelial cells (CS-Endo) and primary human CSF epithelial cells (CS-Epi) were purchased from both Sciencell (Carlsbad, CA, United States) and Creative Bioarray (Shirley, NY, United States). CS-Endo were maintained in 5% fetal bovine serum (FBS) endothelial cell growth media (Sciencell, Carlsbad, CA, United States). CS-Epi were maintained in epithelial cell medium (Sciencell, Carlsbad, CA, United States) with only 1% FBS. After receipt from the vendor, primary CS-Endo and CS-Epi were thawed, expanded for three passages, and banked as frozen stocks in complete media supplemented with 10% dimethyl sulfoxide (DMSO). A single vial of cells was then thawed and allowed to recover for three to 4 days before being seeded into the BCSFB device.

### Organ chip loading

Before endothelial cells were introduced into the lower vascular chamber, the BCSFB device was coated with 2 μg/cm^2^ fibronectin in the lower chamber and with poly-L-lysine (2 μg/cm^2^) in the upper chamber (Sciencell, Carlsbad, CA, United States). The chip was then placed in a 37 °C incubator overnight.

The seeding of BCSFB organ chips with CS-Endo in the lower chamber and CS-Epi in the upper chamber was accomplished in two steps. On day 0, CS-Endo cells were loaded into the lower vascular network and chamber at a density of (8–10) × 10^6^ cells/mL. The chip was then inverted and placed in the incubator overnight, during which time the cells were in the media trapped in the channels and chamber but without perfusion. This process facilitated the attachment of the cells to the vascular side of the porous barrier membrane, which will be the underside of the membrane during barrier tests. As the second step, on day 1 the device was removed from the incubator and returned to its original orientation, with the reservoirs now on top as shown in Figure 1, with the endothelial cells adherent to the underside of the barrier membrane. CS-Epi cells were loaded into the upper CSF chamber at a density of (8–10) × 10^6^ cells/mL and given 2 h for attachment, which was assessed with phase microscopy. Once the epithelial cells were attached, both inlet reservoirs were filled, and gravity flow was initiated for both sides of the membrane. Media was then perfused through both chambers at a rate of ∼2 μL/min, although the rate will change over time due to the gravity perfusion of the device and the continuous changes in the heights of the inlet and outlet reservoirs. Primary CS-Endo were perfused with endothelial cell growth media, and the chamber containing CS-Epi was perfused with epithelial cell medium with no FBS.

### Permeability analysis

Removal of waste media and addition of new feeding media occurred every 24 h 200 μL of outlet effluent was collected from both sides at the same time every day for evaluation of barrier function, as the inlet media of the vascular chamber contained a fluorescently labeled dextran whose concentration we could measure in the outlet media from the CSF chamber to see how much of it crossed the barrier. We used fluorescently labeled 3 kD Alexa Dextran 680 (Thermo Fisher Scientific, Waltham, MA, United States) that was added to the endothelial media and present for the entire duration of the experiment to quantify barrier permeability, which was calculated as in our previous BBB work (Brown et al., 2020).

### Immunofluorescence

Immunofluorescence labeling of junctional proteins was carried out as previously described (Brown et al., 2016), with specific changes listed here for staining within a gravity device. All media from inlet and outlet chambers was removed and the chips were washed by injecting 100–200 µL 1X PBS (Thermo Fisher Scientific, Waltham, MA, United States) by syringe, then filling the inlet chambers with 5 mL of 1X PBS. Gravity flow of 1X PBS for at least 15 min at room temperature was allowed. After removal of all PBS from the inlet and outlet chambers, cells were fixed by injecting 100 µL undiluted fixation solution (BD Biosciences, cat#554714) into the chambers by syringe, followed by incubation for 15 min at room temperature. After fixation, washing was again performed via injection of 100–200 µL PBS into each chamber by syringe, followed by incubation for 10–15 min at room temperature. Then all fluid was removed from the outlet chambers and the inlets were filled with 3–5 mL of 1X PBS +2% goat serum (Thermo Fisher Scientific, Waltham, MA, United States) and incubated overnight at 4 °C to block. Next, all fluid was removed from the inlet and outlet chambers and 100 µL of a primary antibody solution (1:50 ZO-1, occludin, and VE cadherin (33-9100, 33-1500, PA5-17401 Thermo Fisher Scientific, Waltham, MA, United States), was injected by syringe into each chamber to incubate overnight at 4 °C. On the following day, 100–200 µL 1X PBS +2% goat serum was injected into each chamber by syringe to wash unbound antibody. Finally, all fluid was removed from the inlet and outlet chambers and 100 µL of secondary antibody solution was injected into each chamber by syringe and allowed to incubate at least 2 h at room temperature or overnight at 4 °C. On the last wash, 100–200 µL PBS was injected into each chamber by syringe to wash unbound antibody. In the final wash step, NucBlue (Thermo Fisher Scientific, Waltham, MA, United States) Ready Probe Reagent (2 drops per mL) was included to stain nuclei. 1 mL of PBS-S was added to the inlet and outlet chambers to minimize drying.

## Preparation of PEG stock solutions

Linear methoxy polyethylene glycol propylamine (PEG) of various molecular weights (5-kDa, 20-kDa, 40-kDa) were obtained from NOF Corporation America (White Plains, NY, cat# MEPA-50H, MEPA-20T, MEPA-40T, respectively). PEG stock solutions were prepared fresh or stored short-term at 4 °C. 5, 20 and 40-kDa PEG (solid) were dissolved in warm cell endothelial cell medium at concentrations ranging from 10% to 50% (w/v). Solutions were sonicated and warmed to 37 °C–50 °C to facilitate dissolution. All stock solutions were filter sterilized (0.22 µm) prior to use.

### PEG treatment

PEG stock solutions were diluted in pre-warmed complete endothelial cell culture medium to achieve final working concentrations of 0.5–2 mM, depending on experimental requirements. This dose range was based on previous animal studies as well as limited human clinical data on potential blood concentrations (Niven et al., 1994) and as positive control for inducing vacuolation in an *in vitro* system (Fang et al., 2022). Dosing took into consideration the human rate of CSF turnover, which is between 3 and 4 times within a 24-h period (Benarroch, , 2016). For treatment, existing cell culture medium was aspirated and replaced with PEG-containing medium on the vascular side only on day 1 after co-culture had been established. Control chips received vehicle-only treatments (e.g., PBS or media without PEG). Cells were incubated at 37 °C with 5% CO_2_ for up to 15 days.

### Assessment of cellular responses

Following PEG treatment, cell viability was assessed using a live/dead cell imaging kit (Thermo Fisher, Waltham, MA, United States). Barrier integrity between the epithelial and endothelial layers was evaluated using paracellular tracer permeability assays (e.g., Alexa 680-Dextran).

### Immunofluorescent staining of PEG

Chips were fixed in 4% paraformaldehyde for 20 min, permeabilized with 0.1% Triton X-100 for 20 min, and blocked in 5% normal goat serum overnight at 4 °C. Chips were incubated overnight at 4 °C with primary anti-PEG antibody (dilution 1:200 in blocking buffer) # NBP2-77399 (Novus, St. Charles, MO, United States), washed three times with PBS, and incubated for 1 h with Alexa Fluor-conjugated secondary antibodies (1:500). Nuclei were counterstained with DAPI (1 μg/mL).

### Reproducibility

Reproducibility was evaluated two ways, the first of which was independent chip experiments (referred to as “runs”) indicating the robustness of the platform in terms of reproducible results. The second assessed the biological phenotype via independent cohorts representing unique cell lots.

### Polyethylene glycol quantification

Polyethylene glycol (PEG) concentrations were quantified using a competitive PEG ELISA Kit (ab215546, Abcam, Waltham, MA, United States) according to the manufacturer’s instructions. Briefly, all reagents, standards, and samples were equilibrated to room temperature prior to analysis. Samples were diluted in the supplied antigen/antibody diluent buffer, as required, to ensure that measurements fell within the assay’s dynamic range (2–200 ng/mL). PEG concentrations were determined by interpolation from a standard curve generated using a four-parameter logistic (4 PL) regression model. Because this is a competitive ELISA, absorbance is inversely proportional to the concentration of PEGylated molecules present in the sample. All samples and standards were analyzed in duplicate, and calculated concentrations were corrected for sample dilution factors where appropriate ELISA quantified PEG present in the epithelial chamber culture medium, not cell-associated PEG. Therefore, the measured signal reflects the fraction of PEG that reached the epithelial chamber during the experimental period.

### PEG puncta quantification

Imaging of the BCSFB chip was performed using the Revvity Opera Phenix Plus High-Content Imaging System, equipped with a custom chip holder and a ×40 air objective (NA 0.75, long working distance). A 2 mm^2^ area of the membrane was imaged using a 24-plane z-stack with 2.5 μm spacing, covering a total depth of 57.5 μm. This range ensured sufficient resolution both above and below the membrane.

Fluorescence signals were captured in two channels: the target marker (PEG) was imaged using the Cy3 channel (excitation: 561 nm; emission: 570–630 nm) with a 1000 m exposure time and 90% laser power. The nuclear stain (Hoechst) was imaged in the DAPI channel (excitation: 405 nm; emission: 435–480 nm) using the same exposure and laser settings.

Image analysis was conducted using a custom pipeline developed in Revvity Harmony 5.2 software, named “BCSFB_Spot_Analysis_v1.” The algorithm applies separate maximum intensity projections for the regions above and below the membrane. For each projection, the pipeline utilizes the “Find Nuclei,” “Find Cytoplasm,” and “Find Spots” modules, configured based on Revvity technical recommendations and optimized for BCSFB chip morphology.

Separate analyses were generated for the regions above and below the membrane. Output data at the “Well Results” level were extracted for each chip, then aligned and merged to generate a single dataset per chip. These datasets were subsequently aggregated across chips to generate results for the full experimental cohort.

The distribution of discrete PEG foci/spots in cells, referred to herein as “puncta,” was quantified by calculating the ratio of spot counts above (i.e., the epithelial layer) versus below (i.e., the endothelial layer) the membrane (Top/Bottom). These ratios were then compared across experimental conditions to assess differential localization of puncta, as well as looking at puncta diameter as an indication of quantity.

### Transport assay

We assessed active transport across our hBCSFB using two compounds, chloramphenicol (Cho) and ceftriaxone (Cef). A stock of Cho (Sigma, St. Louis, MO, United States) was made: 50 mg/mL in ethanol working stock was diluted in cell culture media down to 5 mg/mL. Cef (Sigma, St. Louis, MO, United States) was made: 100 mg/mL in water working stock was diluted in cell culture media down to 50 mg/mL.

Chips were cultured for 5 days to allow barrier function to be assessed, and on the sixth day either Cef [50 mg/mL] or Cho [5 mg/mL] was added to the inlet media of the vascular chamber only. After 24 h, outlet media from both the vascular and the CSF chamber was collected and sent to Vanderbilt University’s Mass Spectrometry Core for quantitation of Cef and Cho, both of which were already shown to be reasonably stable in our media in previously conducted pilot studies. By taking the concentration of the initial inlet media and comparing it to the concentration found in the outlet media, we were then able to calculate a percent transport of the compound across the BCSFB.

For quantifying transport, standards for Cef and Cho were ordered from Millipore Sigma, and a 5 mg/mL standard solution was prepared for each. From this solution a serial dilution was made to create a standard curve ranging from 1 to 500 μg/mL. The samples were diluted 10x in mobile phase A before being run on a Thermo Quantum Ultra AM triple-quad mass spectrometer with a Waters Acquity HPLC system. The samples were injected onto an Agilent Poroshell 120 EC-C18 2.7 µM 3.0 × 50 mm column starting at 100% Mobile Phase A (0.2% formic acid 95% H_2_O/5% ACN), holding for 1 min before going to 100% Mobile Phase B (0.2% formic acid 5% H_2_O/95% ACN) over 6 min, then holding for another minute before returning to the starting conditions for 2 min. The mass spectrometer was monitoring for Cef in positive mode with SRM 555.1 to 112 and Cho in negative mode with an SRM 322 to 258, with the data collected analyzed using LCQuan from Thermo Fisher.

Media controls were used using ion mobility-mass spectrometry (IM-MS) with and without 1% BSA, and there was no significant protein binding observed (<3%) Cho and Cef perfusate was diluted to 5 and 50 mg/mL respectively, resulting in a <5 mOsm.

### Statistical analysis

Statistical analyses were performed using GraphPad Prism (version 11.02; GraphPad Software, San Diego, CA, United States). Data are presented as mean ± standard error (SE) unless otherwise indicated. Each biological replicate represents an independent microphysiological device prepared and analyzed separately. For comparisons between two groups, an unpaired two-tailed Student’s t-test was used for normally distributed data with equal variance. When assumptions of normality were not met, the nonparametric Mann-Whitney U test was employed. Comparisons involving three or more groups were analyzed using one-way or two-way analysis of variance (ANOVA), as appropriate, followed by Tukey’s multiple-comparisons test. If normality assumptions were violated, the Kruskal–Wallis test with Dunn’s multiple-comparisons correction was used. Given the limited sample sizes, statistical conclusions were interpreted cautiously, with emphasis placed on effect size, consistency across independent experiments, and biological relevance in addition to P values. All tests were two-sided, and P < 0.05 was considered statistically significant.

## Results

### Platform layout and design

To address the critical absence of NAMs of the blood-cerebrospinal fluid barrier (BCSFB), we set out to design and fabricate a two-chamber organ chip capable of independent perfusion on both sides of the barrier to mimic both blood and CSF flow and allow easy sampling of effluent from each side for real-time functional assays (Figure 1A). These two chambers are separated by a porous membrane to allow epithelial-to-endothelial cell-to-cell contact while still providing a scaffold for cell adhesion and organization. As space and throughput are always a consideration in an *in vitro* assay, we designed the chips to be compatible with either gravity flow or pump perfusion systems. Thus, although our BCSFB chip retains punch ports for pressure-fit Tygon tubing to allow connection to a pump perfusion system, we also designed large, low-profile reservoirs bonded to the chip to provide continual perfusion for at least 24 h (Figure 1B). The vascular/blood side of the chip contains a splitter network that ensures uniform and higher shear forces on the endothelial cells. The CSF side lacks this splitter network to increase the channel cross-sectional area, reduce shear forces, and thus better recapitulate the physiological conditions that these cells would normally experience. The scaffold and perfusion were designed to allow the cells to grow and function within a more accurate physiological range than is achievable in two-dimensional cell culture on flat plastic. With this design we have created a platform that more closely recapitulates the human BCSFB when compared to traditional cell culture systems, including Transwell™ and organoids.

### Barrier integrity and permeability

**FIGURE 2:**
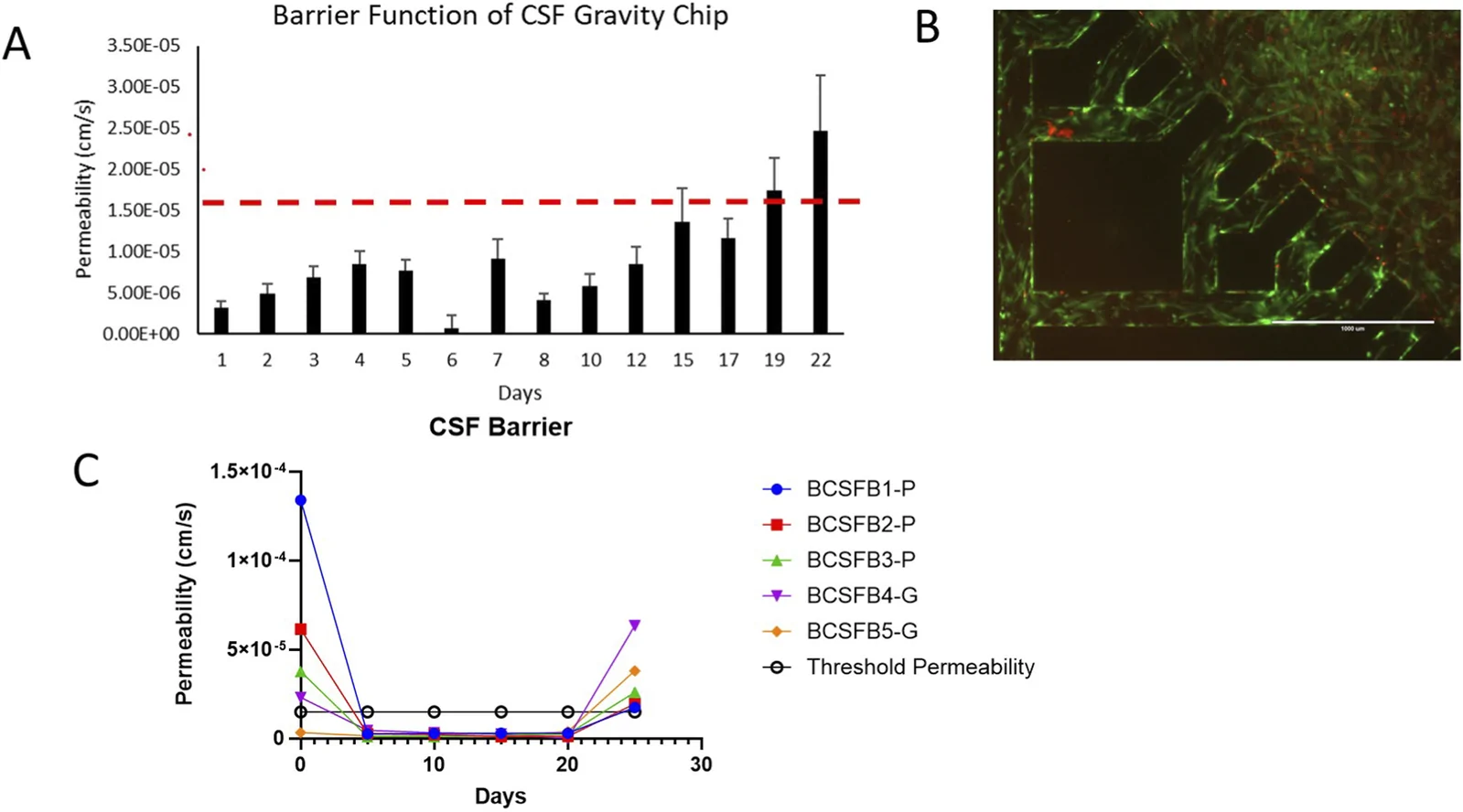
Permeability of BCSFB MPS. **(A)** Over the course of 22 days in culture, the permeability of 3kD fluorescent dextran showed that the majority of chips maintained good barrier function until day 15 (N = 15). Dashed red line indicates the maximum permeability acceptable for barrier functional assays. **(B)** Live/dead imaging of a BCSFB chip at day 15 showing both epithelial and endothelial cells; *in vivo* green = alive; red = dead. **(C)** Comparison of devices perfused by gravity (G) versus a syringe pump (P), showing no difference in barrier function as a result of perfusion method.

Having designed and tested the platform for cell attachment, viability, and perfusion, we turned to evaluation of physiological performance. The organ chips were loaded and the permeability measured as described above. From previously published values for BCSFB permeability for other fluorophores and molecules, we estimated that the human barrier permeability for our dextran of 3kD should not be greater than 1.55E-5 cm/s (Dinner et al., 2016; Schroten et al., 2012; Schwerk et al., 2012; Su et al., 2015); thus this was established as the cutoff for chips that were performing well in terms of their barrier function. Combining data from four separate independent chip experiments of the BCSFB (N = 15), we found that 14 out of the 15 chips maintained their barrier function for 15 days *in vitro* under gravity perfusion (Figure 2A). Overall health and viability of the chip were further confirmed using live/dead calcium dye imaging that showed good cell viability throughout the experimental time course (Figure 2B). We then compared gravity perfusion to syringe pump perfusion, with no real barrier difference seen during the optimum culture window and no significant extension of the model’s viability with syringe pumps (Figure 2C). We also show that this restricted permeability is a result of cellular function not just the physical properties of the chip (Sup Figure 1). To date, this platform has one of the longest (at 15+ days) and most reproducible physiologically relevant barrier functions for an *in vitro* system, arguing that it will be a valuable tool for further research on the BCSF barrier.

**FIGURE 3:**
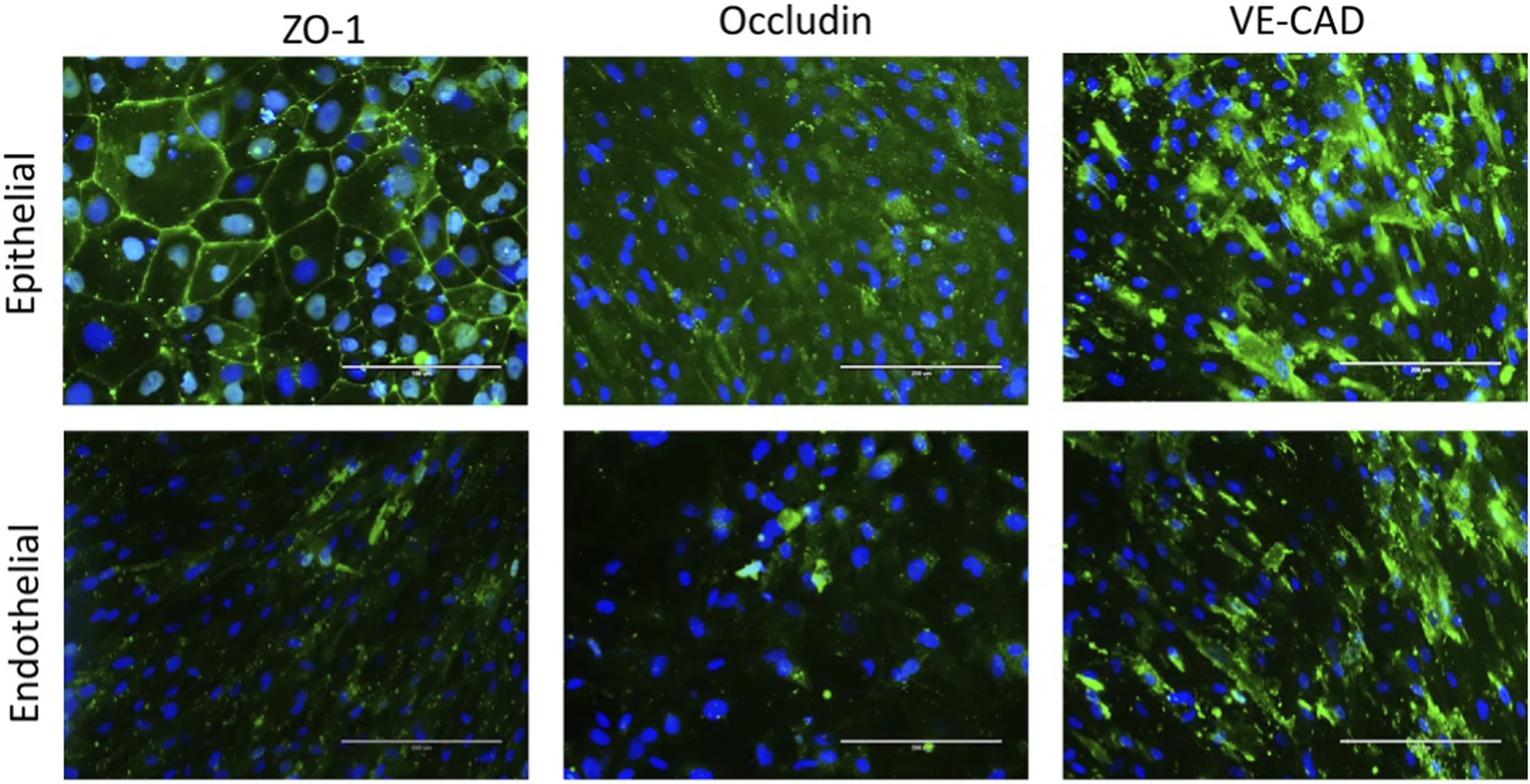
Junctional protein expression in BCSFB MPS. The expression of three different junctional proteins (ZO-1, occludin, and VE-cadherin) demonstrates that the epithelial layer has good junctional protein expression, while the endothelial layer has the expected reduced expression of these markers in the blood-CSF barrier. Measurement bar for epithelial ZO-1 is 100 μm, all others 200 µm.

The next goal was to establish that the functional permeability observed in the chips correlated with the appropriate differential expression of key junctional proteins for the vascular and CSF sides of the BCSFB. As previously stated, unlike the blood-brain barrier, it is the epithelial cells that more prominently express the junctional proteins of the BCSFB (Kratzer et al., 2020). In a panel of junctional proteins including ZO-1, occludin, and VE-cadherin, we found that our BCSFB chips did indeed express these junctional markers preferentially in the epithelial layer versus the endothelial layer, as assessed via fluorescent imaging of antibody-labeled junctional proteins (Figure 3). This immunofluorescent imaging of the junctional proteins, combined with the permeability data discussed earlier, strongly argues that functional outcomes from this three-dimensional co-culture system under continuous perfusion are the result of appropriate physiological expression of the junctional proteins.

### Dose and molecular weight-dependent PEG sequestration

**FIGURE 4:**
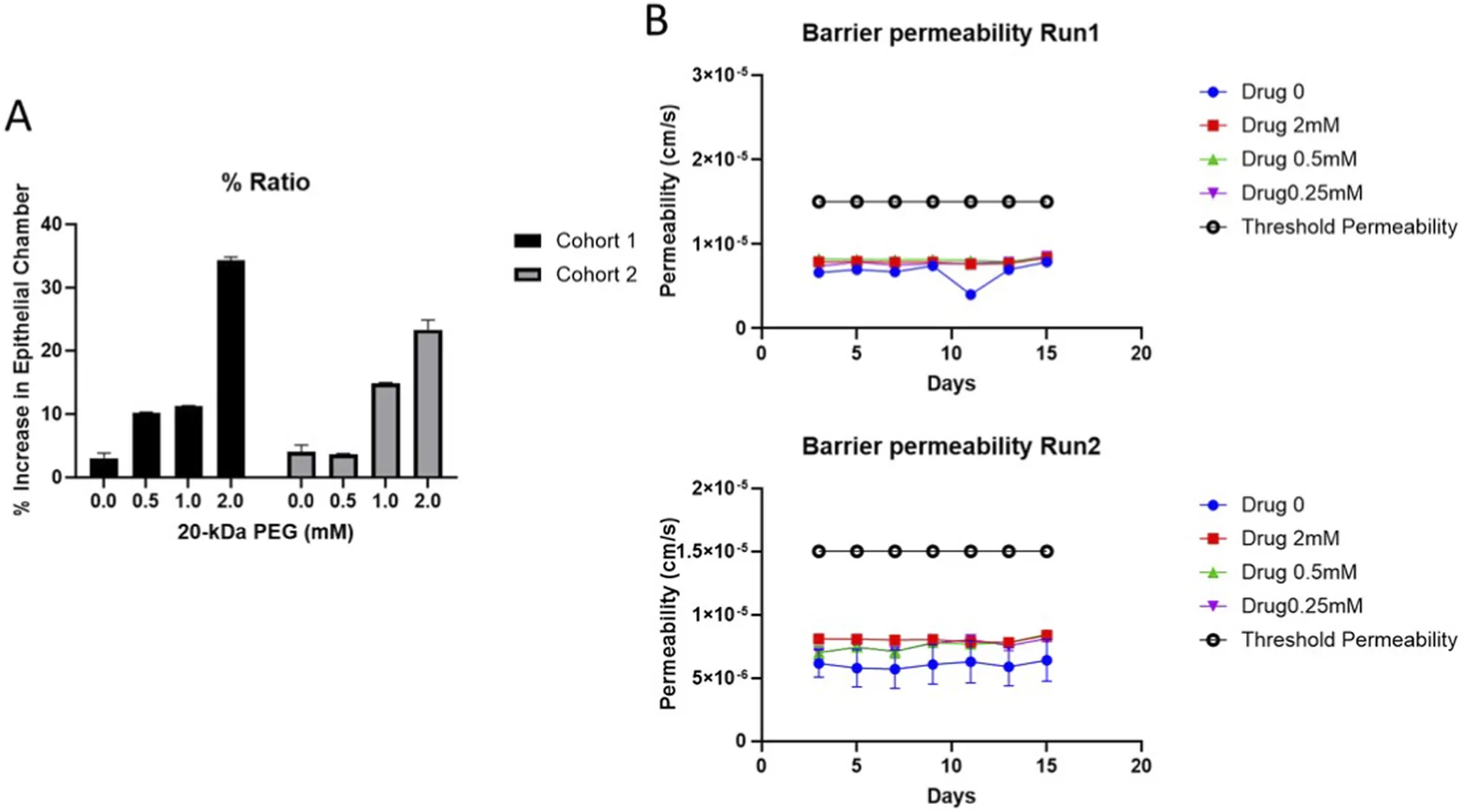
BCSFB MPS sequestration and permeability after exposure to 20-kDa PEG. **(A)** Measurement of PEG accumulation in the epithelium as a function of increasing dose, showing a reproducible dose-dependence. **(B)** A demonstration of the uniformity of barrier permeability over the 15-day time course of PEG exposure in two independent cohorts of BCSFB MPS chips.

Once we had a platform that met the original benchmarks of appropriate barrier function and junctional protein expression, we wanted to further establish the functional effectiveness of our platform by evaluating its ability to mimic non-specific transport in a dose-dependent manner using PEG, which has been demonstrated to be transported at higher molecular weights via endocytosis (Wang et al., 2020). Using three different doses of a 20 kDa molecular weight PEG, we were able to show a consistent dose-dependent accumulation in the choroid plexus epithelium of the BCSFB (Figure 4A). Although the exact percent sequestration to the epithelium from the vascular media had a small variation between the two separate experimental independent chip experiments, the dose-dependent trend was highly consistent between the two independent studies, indicating a high degree of reproducibility. This increasing amount of PEG cannot be accounted for by changes in permeability function, as permeability remained consistent over the time course of the experiment and was unaffected by drug concentration (Figure 4B). We were interested in evaluating any potential impact of PEG accumulation on BCSFB function because PEG-induced accumulation and vacuolation in the choroid plexus epithelium induced by some PEGylated biopharmaceuticals have been of considerable concern to pharmaceutical scientists and regulators (Irizarry Rovira et al., 2018; Ivens et al., 2015). The results of this experiment indicated that accumulation of 20 kDa PEG did not impair barrier function.

**FIGURE 5:**
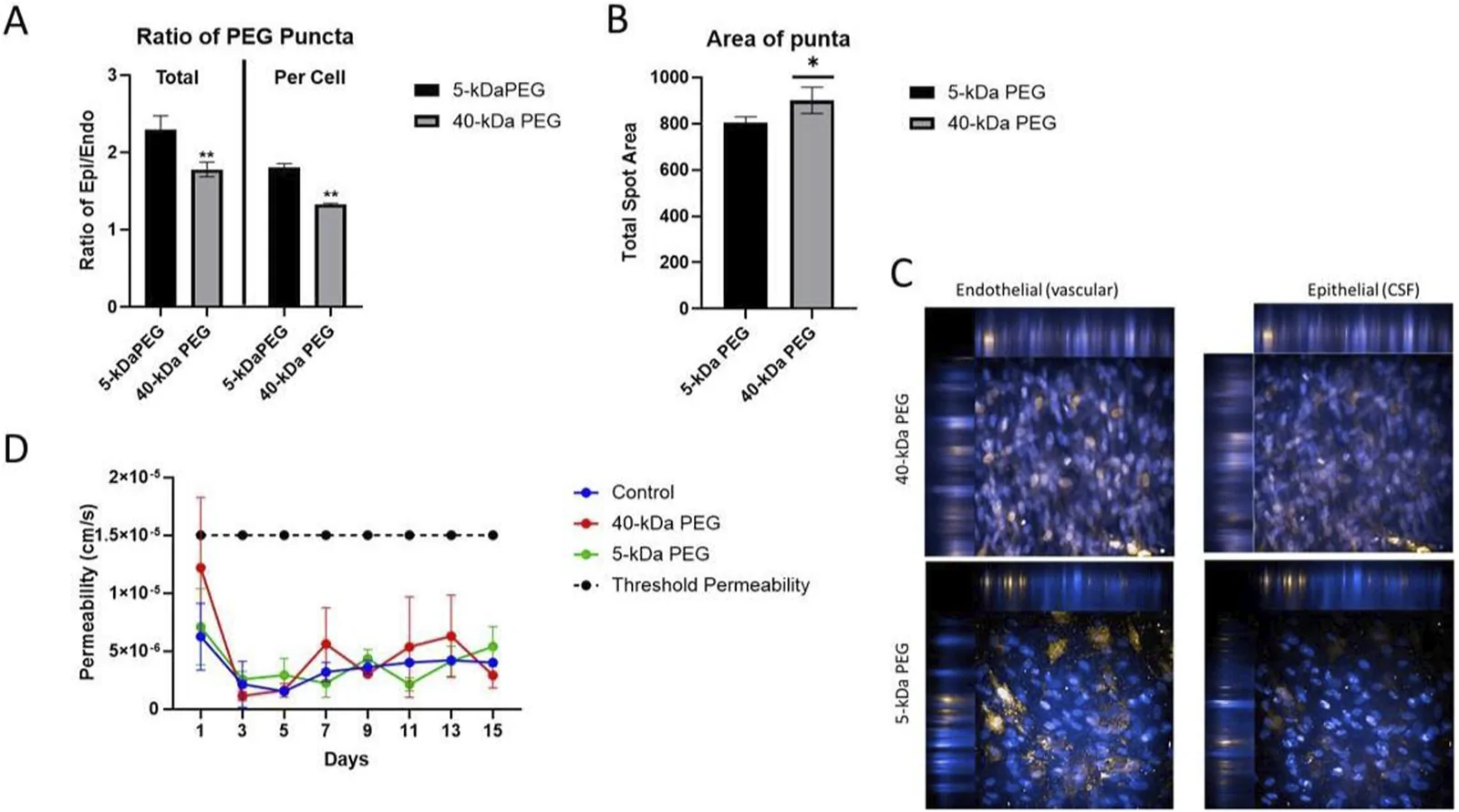
BCSFB MPS quantification of PEG sequestration as a function of molecular weight. **(A)** Quantification of antibody labeled PEG as a ratio of the fluorescent intensity from the epithelial side of the BCSFB to that from the endothelial side. For both total and per cell, significantly less PEG was observed in the epithelium at the higher molecular weight than the lower molecular weight (p = 0.01 N = 5). **(B)** Quantification of the area of antibody labeled PEG puncta shows a significant increase in puncta area for higher molecular weight PEG (p = 0.05 N = 5). **(C)** Immunofluorescent images showing the antibody labeling of PEG within the BCSFB. **(D)** BCSFB permeability was unaffected by exposure to either high or low molecular weight PEG and remained stable over the 15-day time course.

In addition to dose-dependent sequestration, we looked at the influence of PEG molecular weight and its effects on vacuole sequestration and barrier function in the BCSFB. It has been established in animal models that the PEG moieties of PEGylated biopharmaceuticals induce vacuolation and accumulation of PEG in the choroid plexus epithelium and that larger molecular weight PEGs may induce more or larger vacuoles (Irizarry Rovira et al., 2018). However, it has not been known if accumulation of the larger PEGs has an impact on BCSFB function and whether this phenomenon occurs in human tissues. Here we report that although smaller molecular weight PEGs cross more readily across the BCSFB into the choroid plexus epithelium (N = 5 p ≤ 0.01) (Figure 5A), the size of individual PEG puncta as measured by their area is greater with higher molecular weight PEG (N = 5 p ≤ 0.05) (Figure5B). Representative images of PEG immunofluorescence on the endothelial and epithelial sides of chips are shown in Figure 5C. Interestingly, neither the small nor the large molecular weight PEG has any significant effect on barrier permeability (Figure 5D), indicating that accumulation of PEG did not impair barrier function at molecular weights tested.

### Selective transport

**FIGURE 6:**
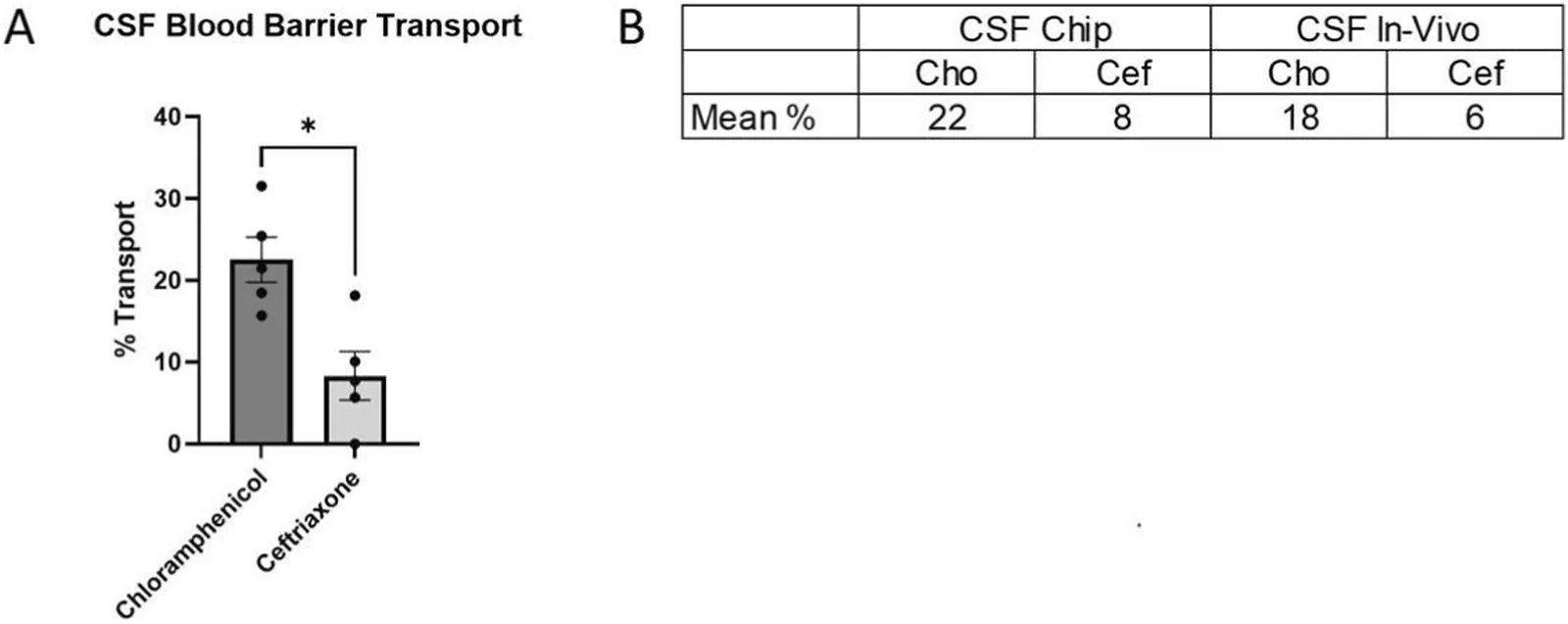
Selective percent transport of Cho and Cef in BCSFB MPS. According to human subject data, Cho is well transported through the BCSFB, while Cef is not. **(A)** The average percent transport as determined by mass spectrometry (N = 5 p = 0.03). **(B)** A comparison of percent transport between our novel MPS platform and human subject transport efficiency (from clinical data) demonstrated that not only do our chips show the differential transport expected of these two compounds, but also that their percent transport is consistent with that of the human subjects.

Having established junctional protein expression, appropriate physiological barrier permeability, and dose- and size-dependent transition to the epithelium, the next parameter we sought to investigate was that of selective transport. To provide this challenge, we identified from the literature two compounds for which excellent human data exist in terms of transport efficiency across the BCSFB (Nau et al., 2010). We intentionally selected one compound with exceedingly poor transport, ceftriaxone (Cef) (Le Turnier et al., 2019), and one with relatively good transport, chloramphenicol (Cho). Reduced CSF deposition of cef is attributed to organic anion transport (OAT)-mediated pathways and P-glycoprotein (P-gp) efflux mechanisms that facilitate clearance from the CSF and limit accumulation within the CNS (Takeda et al., 2002), while Cho (Ristuccia and Le Frock, 1971) is not subject to this kind of efflux (Nau et al., 2010), thus providing a more rigorous test for our platform’s performance. For each compound, five chips were used in the transport study, and only chips whose permeability was performing at criteria were included in the drug transport evaluation (Supplementary Figure S1). As was predicted from human data, Cho showed significantly higher transport (p = 0.03; N = 5) over ceftriaxone (Figure 6A). Not only did the BCSFB MPS show appropriate transport of the two test compounds, when we directly compared the percent transport across the chip from the vascular side to the CSF side, we found that the percent transport in the *in vitro* chip was well matched to *in vivo* data from human subjects (Figure 6B). (Neal et al., 2021) While it is arguably important for an *in vitro* model of the BCSFB to have the appropriate barrier restrictive properties, this evidence of selective access serves as a necessary benchmark for validation of a more complete platform with wider applicability.

## Discussion

Although a great deal of work has been done in the world of MPS platforms on generating blood-brain barriers, to date few if any validated MPS organ chips are available for the BCSFB (Dabbagh et al., 2022). Some of the challenges of creating this *in vitro* model arise from the fact that at least in traditional cell culture models, human primary cells do not always express the proteins that would be expected (Tenenbaum et al., 2008). Several possible causes of this failure in expected protein expression in 2D models include the possibility of a physiological need for shear stress from directional flow to properly polarize the endothelial and the epithelial cells that contribute to barrier function (Frost et al., 2019; Lechuga et al., 2024); the possibility that perfusion of one or both sides of the BCSFB may wash away inhibitory growth factors or cytokines that adversely affect cell phenotype in 2D culture on plastic (Shin et al., 2019); or that simple models fail to recapitulate BCSFB function because of the lack of co-culture and the associated cues that define apical and basolateral exposure (Hakvoort et al., 1998; Redzic, 2011). Another challenge of 2D models on plastic is that the CSF vascular cells cannot be grown in serum-free media without experiencing serum starvation (Dabbagh et al., 2022; Klas et al., 2010), while the CSF epithelial cells need an absence of serum to express junctional proteins appropriately, creating an inherent conflict for any co-culture system that cannot support two different medias on opposite sides of some sort of barrier membrane (Dabbagh et al., 2022). Organoids have several advantages, including self-organization, ease of generation, and good batch scalability. However, these organoids lack fluidic shear stress or media washout, have no true vascular-epithelial interface, and often have high batch-to-batch variability with a restricted control of the microenvironment for media composition and intra-organoid gradients, as well as difficulty in real-time readouts for drug permeability and barrier function (Zhao et al., 2021). It is known that maintenance of normal physiology and organ function are dependent on the complex interaction of all organs in the body and that dysfunction in one organ can impact the function of other organs (Che et al., 2026). Thus, it is not surprising that the BBB and BCSFB work closely with one another to provide barrier function to the CNS. Although future work could create a complex MPS that incorporates the BBB and BCSFB, by creating individual MPS of the BBB and the BCSFB we can isolate each system and start to interrogate their function before increasing the complexity by adding multiple systems to evaluate their contribution. It was with these challenges and requirements in mind that we set out to develop an *in vitro* MPS to model the BCSFB using human primary cells.

Traditional two-dimensional cell culture often fails to achieve the appropriate expression of junctional proteins as well as cell morphology (Tan et al., 2021), so we felt it critical to establish that this was a deficit in the way the cells were being cultured, not in the cells themselves. By creating a NAM that has flow with the possibility of shear-stress cell polarization, the possibility of factor/cytokine washout, appropriate co-culture signaling that might establish apical and basal orientation, and the ability to have serum-containing media on the vascular side and serum-free media on the epithelial side, we were able to validate with immunohistochemistry the appropriate cell morphology and junctional protein expression of the primary cells in use in the chip, demonstrating that the environment of cell culture directly affected the physiology of the cells in use. We do acknowledge that given the thickness of the device which allows for the gravity perfusion and therefore the higher throughput and better containment does reduce the quality of the imaging within the device however we think this is an acceptable trade off given the model’s other strong points. We also have not ruled out the possibility that perfusion of one or both sides of the BCSFB may wash away cytokines or inhibitory growth factors that adversely affect cell phenotype in 2D culture on plastic (Shin et al., 2019; Xin et al., 2025).

As with any novel platform, it was critical to provide a variety of tests for validation and to show that these parameters were reproducible in multiple cohorts. As the quantification of barrier properties was a significant part of chip validation, we ran four separate cohorts, each loaded with its own set of cells that came from at least two different lots and vendors, and we were able to demonstrate, over multiple cohorts, physiologically appropriate barrier function (Solar et al., 2020) for at least 15 days *in vitro*, indicating a robust and highly reproducible platform for BCSFB evaluation.

PEG-induced accumulation and vacuolation within the choroid plexus epithelium has been observed in multiple *in vivo* studies of PEGylated biopharmaceuticals, particularly those with higher molecular weight forms (Irizarry Rovira et al., 2018; Ivens et al., 2015). PEG alone does not engage in receptor-mediated transport pathways (Xu et al., 2013); consequently, its passage across the barrier reflects passive transport processes. This property makes PEG particularly well suited for directly assessing baseline barrier behavior, independent of active or receptor-driven transport mechanisms. Despite reports that high molecular weight PEGs (>10 kDa) do not significantly cross the BCSFB (Fukuda et al., 2021), the presence of PEG within choroid epithelial cells suggests that there is selective sequestration and limited or no transcytotic transport out of the epithelial cells and into the CSF (Schoenbrunn et al., 2022). This phenomenon appears to be influenced by both the dose and molecular weight of PEG (Fang et al., 2022; Fornasari, 2025). Specifically, as the molecular weight of PEG increases, so does its retention within the epithelium, suggesting a threshold beyond which clearance or further translocation becomes inefficient or obstructed. Higher systemic doses further intensify this effect, likely overwhelming normal endocytic recycling or degradation pathways, leading to vacuolization. Although PEG is generally regarded as biologically inert, its selective accumulation in epithelial tissues has raised concerns regarding long-term exposure, epithelial stress, and potential barrier dysfunction, especially in therapeutic settings involving repeated administration of PEGylated compounds. Using this MPS platform, we were able to directly assess barrier integrity in terms of permeability and demonstrate that over a 15-day period vacuolation did not result in barrier disruption, an evaluation that was previously not feasible. These findings align with existing data from animal studies involving PEGylated biopharmaceuticals (Irizarry Rovira et al., 2018; Ivens et al., 2015). and with *in vitro* investigations demonstrating that PEG accumulation did not result in cellular dysfunction (Schoenbrunn et al., 2022). These data were the driving force behind using a relatively high concentration of PEG to substantially cause accumulation and vacuolation to investigate its effects on barrier function. It is also important to note that PEG may alter transport expression by osmotic or membrane tension (Malinin et al., 2002; Wang and Zuo, 2018), and while this has not been confirmed in the context of the BCSFB, it could potentially be a valuable direction to investigate with this model in future studies. While further work is needed for consideration of the potential consequences of prolonged exposure and PEG sequestration, this model gives United States tool that will enable additional investigation into the potential effects of PEG or other xenobiotics on the function of the hBCSFB. Furthermore, as an initial test of the platform as an *in vitro* safety assay, we demonstrate that exposure to monomeric polyethylene glycol of various molecular weights does not impact the function of the permeability barrier of the hBCSFB.

In addition to passive restrictive barrier properties, selective access of compounds into the CSF is a critical feature of the BCSFB (Nau et al., 2010; Mila-Aloma et al., 2021). Even though the BCSFB is less restrictive than the blood-brain barrier, it remains a highly specialized and complex interface responsible for maintaining the ionic composition of the cerebrospinal fluid. Na^+^/K^+^-ATPase pumps actively transport Na^+^ into the CSF, establishing electrochemical gradients that drive Cl^−^ transport via SLC12 transporters and HCO_3_^−^ transport via SLC4 transporters, thereby promoting water secretion (Thompson et al., 2022). In addition to regulating ion and fluid balance, the BCSFB mediates selective xenobiotic transport. Molecular movement occurs either transcellularly through transporter-mediated pathways or paracellularly via tight junctions, with efflux homeostasis maintained in part by P-glycoprotein (Katada et al., 2025). Here we show a difference in deposition between compounds that are relatively insensitive to efflux vs. those that are more effective (Nau et al., 2010), creating a clear difference between CSF deposition. The compounds we utilized, chloramphenicol (Cho) and ceftriaxone (Cef), were selected based on clinical data showing that cho was found at higher concentrations in CSF than serum and that cef had poor penetration. Cho lipophilicity would indicate an ability to diffuse along the concentration gradient, which is likely to be a component of its penetration into the CSF; however, to achieve higher than serum concentrations would still require some form of active transport (Nau et al., 2010). To evaluate our platform’s performance in this area of selective access, we were able to show that even with a good restrictive barrier function, Cho had physiologically relevant penetration into the CSF across the barrier, while Cef did not accumulate, and that the percent transport/penetration efficiency of these compounds was comparable to that observed in human subjects (Spector, 1987; van Niekerk et al., 1980). Hence, our NAM model gives us a tool that can be used to investigate and model drug transport across the hBCSFB.

Drug transport across the BCSFB is governed by a coordinated network of passive diffusion and specialized influx and efflux transporters expressed by the epithelial cells of the choroid plexus. While small lipophilic molecules may enter the cerebrospinal fluid (CSF) by passive diffusion, the disposition of many endogenous compounds and therapeutics is strongly influenced by carrier-mediated transport. Members of the solute carrier (SLC) superfamily facilitate the uptake of essential nutrients, including glucose, amino acids, vitamins, and peptides (Hu et al., 2020), while ATP-binding cassette (ABC) transporters such as P-glycoprotein (P-gp/ABCB1), Breast Cancer Resistance Protein (BCRP), and multidrug resistance-associated proteins (MRPs) actively limit central nervous system exposure by exporting xenobiotics and drug metabolites (Qosa et al., 2015). Organic anion transporters (OATs), particularly OAT3, further regulate CSF drug concentrations by mediating the clearance of endogenous metabolites and numerous anionic therapeutics, including many β-lactam antibiotics (Wu et al., 2017). Understanding and modeling these mechanisms is therefore critical for interpreting CNS pharmacokinetics, identifying barriers to effective drug delivery, and developing therapeutics capable of achieving clinically relevant concentrations within the CSF and central nervous system. While we have only validated certain aspects of these transport systems, there is clearly a basis for future research to more fully characterize all potential transport systems within the context of modeling the BCSFB.

## Conclusion

This work demonstrates the importance of physiologically relevant cell culture conditions for appropriate cellular responses in micro-organ modeling. By providing the appropriate structure and media support, we were able to generate an *in vitro* model of the BCSFB that demonstrates four critical components: 1) restrictive permeability from the blood to the CSF (although this permeability is much higher than is seen in the BBB, as is appropriate), 2) appropriate expression of junctional markers on the CSF epithelial side, 3) nonspecific transport and recapitulation of known dose and molecular weight sequestration of PEG in the choroid plexus epithelium, and 4) selective access of compounds from the blood to the CSF. Although further refinement and complexity of our system is possible, our initial work has firmly established this platform as a highly useful and novel tool in our arsenal for investigation of the BCSFB.

## Data Availability

The original contributions presented in the study are included in the article/Supplementary Material, further inquiries can be directed to the corresponding author.

## Supplementary material

SUPPLEMENTARY FIGURE 1 Assessment of device permeability in the presence and absence of cells. Five acellular devices were compared with five devices containing cells cultured for 5 days, a time point at which a functional barrier is expected to be established. Acellular devices provide only the physical resistance of the porous membrane, whereas cellularized devices include the contribution of the formed biological barrier. The presence of cells significantly reduced the permeability of fluorescent dextran across the membrane compared with acellular devices (p = 0.0001, n = 5 per group).

SUPPLEMENTARY FIGURE 2 Permeability of BCSFB MPS. Chips 1 through 5 were treated on day 6 with Cho, while chips 6 through 10 were treated with Cef on day 6. No significant effect of either drug was seen on BCSF barrier function (N = 5).
